# First Molecular Characterization of *Leishmania* Species Causing Visceral Leishmaniasis among Children in Yemen

**DOI:** 10.1371/journal.pone.0151265

**Published:** 2016-03-11

**Authors:** Mohammed A. K. Mahdy, Abdulsalam M. Al-Mekhlafi, Rashad Abdul-Ghani, Reyadh Saif-Ali, Hesham M. Al-Mekhlafi, Samira M. Al-Eryani, Yvonne A. L. Lim, Rohela Mahmud

**Affiliations:** 1 Tropical Disease Research Center, Faculty of Medicine, University of Science and Technology, Sana’a, Yemen; 2 Department of Parasitology, Faculty of Medicine and Health Sciences, Sana’a University, Sana’a, Yemen; 3 Department of Biochemistry, Faculty of Medicine and Health Sciences, Sana’a University, Sana’a, Yemen; 4 Department of Parasitology, Faculty of Medicine, University of Malaya, 50603, Kuala Lumpur, Malaysia; The Ohio State University, UNITED STATES

## Abstract

Visceral leishmaniasis (VL) is a debilitating, often fatal disease caused by *Leishmania donovani* complex; however, it is a neglected tropical disease. *L*. *donovani* complex comprises two closely related species, *L*. *donovani* that is mostly anthroponotic and *L*. *infantum* that is zoonotic. Differentiation between these two species is critical due to the differences in their epidemiology and pathology. However, they cannot be differentiated morphologically, and their speciation using isoenzyme-based methods poses a difficult task and may be unreliable. Molecular characterization is now the most reliable method to differentiate between them and to determine their phylogenetic relationships. The present study aims to characterize *Leishmania* species isolated from bone marrows of Yemeni pediatric patients using sequence analysis of the ribosomal internal transcribed spacer-1 (ITS1) gene. Out of 41 isolates from Giemsa-stained bone marrow smears, 25 isolates were successfully amplified by nested polymerase chain reaction and sequenced in both directions. Phylogenetic analysis using neighbor joining method placed all study isolates in one cluster with *L*. *donovani* complex (99% bootstrap). The analysis of ITS1 for microsatellite repeat numbers identified *L*. *infantum* in 11 isolates and *L*. *donovani* in 14 isolates. These data suggest the possibility of both anthroponotic and zoonotic transmission of VL-causing *Leishmania* species in Yemen. Exploring the possible animal reservoir hosts is therefore needed for effective control to be achieved.

## Introduction

Human leishmaniasis is a vector-borne disease caused by more than 20 *Leishmania* species in 98 countries on five continents [1]. The disease is categorized as one of the most “neglected tropical diseases'' and has a strong and complex association with poverty [2,3]. Approximately 350 million people are at risk of leishmaniasis, with a yearly incidence of about 0.5 million cases of visceral leishmaniasis (VL), which is responsible for 59,000 deaths annually and losses of 2,357,000 disability-adjusted life years (DALYs), ranking leishmaniasis the ninth in a global analysis of infectious diseases [2]. The disease is caused by two closely related species of the *Leishmania donovani* complex: *L*. *donovani* that is mainly considered to be anthroponotic and *L*. *infantum* that is considered to be zoonotic, with dogs being the main reservoir hosts [4–6]. In the Eastern Mediterranean Region (EMR) of the World Health Organization (WHO), *L*. *donovani* causes anthroponotic VL in Somalia and Sudan [7,8] and is a probable cause of anthroponotic VL in Yemen and Saudi Arabia [6]. On the other hand, *L*. *infantum* causes zoonotic VL in several countries of the EMR, including Yemen [6]. VL is the most severe systemic form of leishmaniasis that is characterized by persistent fever, pancytopenia and organomegaly and is usually fatal if left untreated, particularly among children. Moreover, anti-leishmanial drugs are toxic and given parenterally for long periods [4,9].

In Yemen, leishmaniasis is a major public health problem with a nationwide distribution and is responsible for about 60% of DALYs lost due to tropical diseases prevalent in the country [10]. However, a few studies have been published on VL among pediatric patients from endemic areas of the country. Diagnosis of VL is routinely based on the microscopic examination of bone marrow aspirates together with clinical diagnosis and hematologic investigations [11], which does not enable species identification. This in turn, limits the possibility of developing an effective VL control strategy. A high seroprevalence rate (34.7%; 99/285) of antibodies to *Leishmania* species was reported among schoolchildren from areas endemic with infantile VL in Sana’a and Hajjah governorates [12]. In addition, natural infection of feral dogs from the study areas with *Leishmania* species (50%; 8/16) was also documented for the first time [12]. Haidar et al. [13] reported that the clinical presentation of VL among children in Hajjah governorate, north of Yemen, is similar to that of the Mediterranean type. In Aden, south of Yemen, Abdul Hamid and Gobah [11] reported fever, hepatosplenomegaly and pancytopenia as the most common clinical and hematologic manifestations of VL among pediatric patients. In central Yemen, VL was reported to be the least prevalent form of leishmaniasis, being responsible for 3.3% of cases [14]. However, children and women are at highest risk for contracting the disease [14].

Except for a single study on the molecular characterization of *Leishmania* species causing cutaneous leishmaniasis (CL) in Yemen [15], characterization of parasite isolates was based on isoenzyme analysis. Eight human isolates of *Leishmania* species causing VL in Yemen were analyzed using the isoenzyme method, where seven were identified as *L*. *donovani* and one as *L*. *infantum* [16]. Later, a case of CL in a French visitor to Yemen was also reported to be caused by *L*. *donovani* [17]. However, the isoenzyme method has been recently indicated to be non-robust for the distinction between *L*. *donovani* and *L*. *infantum* compared to molecular discrimination using sequence analysis of ribosomal internal transcribed spacer gene [18]. Therefore, this is the first study conducted to characterize *Leishmania* species causing VL among Yemeni children using the sequence analysis of ITS1.

## Materials and Methods

### Ethical clearance and samples

This study used archived bone marrow smears of 41 pediatric patients admitted in 2010 to Al-Sabeen Hospital, the main referral hospital for women and children in Sana’a, and diagnosed with VL (30 males and 11 females with a mean age of 3.5 years old). The study protocol was approved by the Ethical Committee of University of Science and Technology, Yemen. Permission to use the archived samples was obtained from the hospital management. No informed consent was acquired and patients’ information was maintained confidentially and analyzed anonymously. It should be noted that Al-Sabeen Hospital is a teaching hospital and patients understand that samples may be used in future research. Confirmation of VL was made by the examination of Giemsa-stained smears of bone marrow aspirates, and amastigote density was then determined [19]. *Leishmania* isolates were coded according to the international code system for *Leishmania* isolates following the recommendations of the UNDP/ WHO meeting in Washington, D.C., in 1980 [20].

### DNA extraction, amplification and sequencing

Parasite DNA was extracted from Giemsa-stained bone marrow smears positive for *Leishmania* as previously described [15]. A nested polymerase chain reaction (PCR) was then used to amplify the ITS1 region [15, 21]. PCR products were purified using the QIAquick PCR Purification Kit (QIAgen, Hilden, Germany) according to the manufacturer’s instructions and sequenced using the ABI PRISM^®^ BigDyeTM Terminator v3.0 Ready Reaction Cycle Sequencing Kit (Applied Biosystems, USA) in ABI PRISM^®^ 3700 DNA Analyzer (Applied Biosystems, USA).

### Sequence analysis

Consensus ITS1 sequences of the study isolates were created and multiple-aligned with reference sequences of *L*. *donovani*, *L*. *infantum*, *L*. *major*, *L*. *aethiopica* and *L*. *tropica* retrieved from GenBank using BioEdit software (www.mbio.ncsu.edu/BioEdit/BioEdit.html). Phylogenetic analysis was conducted in MEGA 6 software [22] using neighbor-joining (NJ) method [23], with evolutionary distance calculated by Kimura 2-parameter model [24] and 1000-replicate bootstrap test [25]. For species identification of *L*. *donovani* complex, ITS1 sequences of study isolates were multiple-aligned using ClustalW [26], with ITS1 sequences of previously defined *L*. *infantum* and *L*. *donovani*, representing the different types of ITS1 sequences, and manually analyzed for microsatellite repeats [18]. Sequences from this study have been deposited in the GenBank database under the accession numbers KT751245 to KT751269.

## Results

All Giemsa-stained bone marrow smears were positive for *Leishmania* species amastigotes by light microscopy. The densities of amastigotes were 1+, 2+, 3+ and 4+ in 5, 13, 7 and 16 samples, respectively. One patient had a VL co-infection with malaria. Twenty five (61%; 25/41) isolates were successfully amplified by nested PCR producing the 350 bp amplicon after gel electrophoresis and directly sequenced in both directions. Multiple alignment and analysis of four polymorphic microsatellites in these sequences divided these isolates into two alleles with a single nucleotide polymorphism.

**Fig 1:**
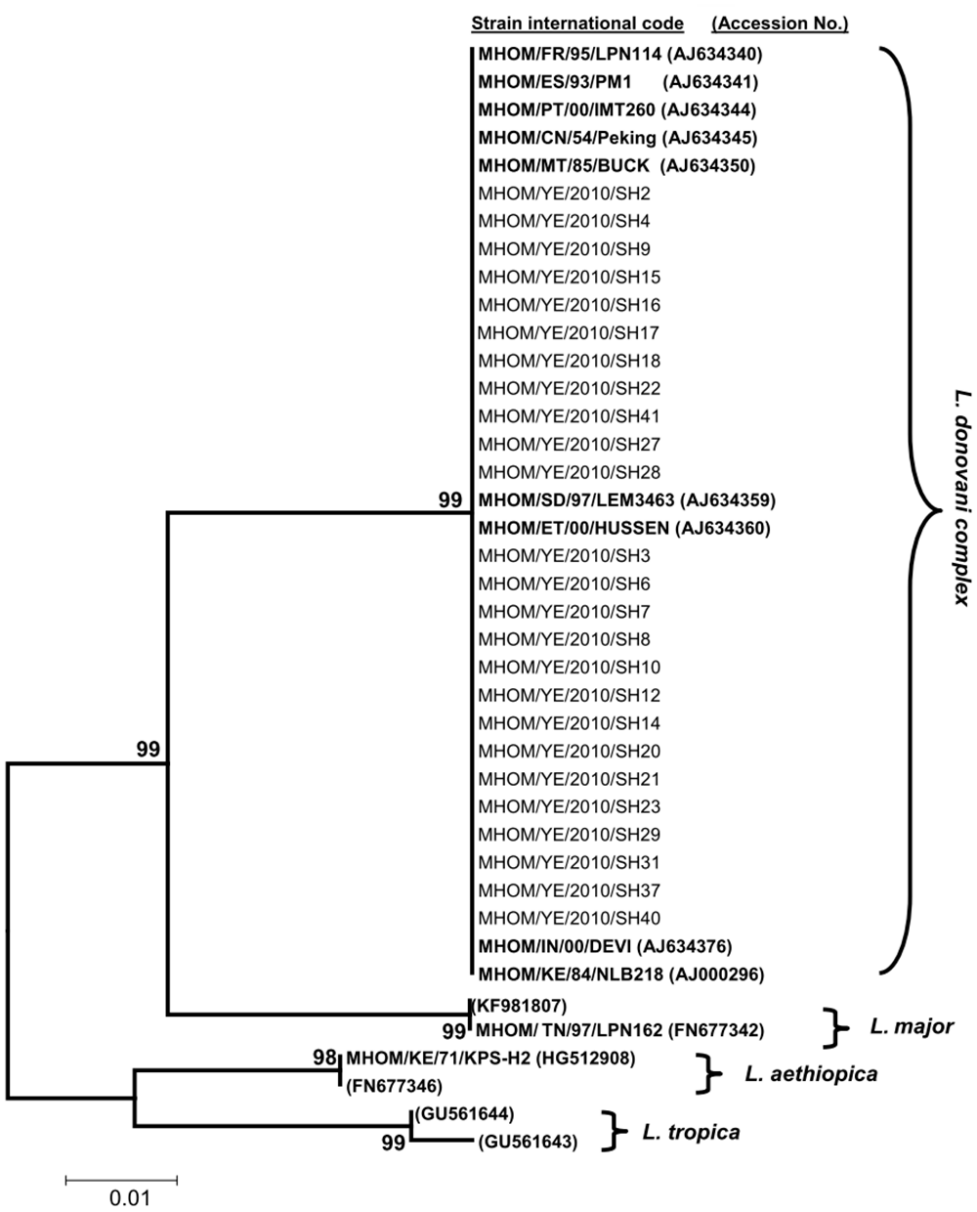
Phylogenetic study of *L*. *donovani* complex causing VL among Yemeni children. Unrooted NJ phylogenetic tree showing the relationships of 25 ITS1 sequences of *Leishmania* species isolates from Yemeni children with VL and sequences representing *L*. *infantum*, *L*. *donovani*, *L*. *major*, *L*. *tropica* and *L*. *aethiopica*. Bold-type represents reference sequences from GenBank. Abbreviations of countries of origib: FR, France; ES, Spain; PT, Portugal; CN, China; MT, Malta; SD, Sudan; ET, Ethiopia; KE, Kenya; IN, India; TN, Tunisia, YE, Yemen.

Phylogenetic analysis using NJ method with reference strains of *L*. *infantum*, *L*. *donovani*, *L*. *tropica*, *L*. *major* and *L*. *aethiopica* placed all *Leishmania* isolates causing VL among Yemeni pediatric patients in one cluster with *L*. *donovani* complex (*L*. *donovani* and *L*. *infantum*) originating from different geographical regions with 99% bootstrap support (Fig 1). Based on the analysis of four polymorphic microsatellite of ITS1 sequences of *L*. *donovani* complex, including the study isolates and reference sequences representing different types of *L*. *infantum* and *L*. *donovani* sequences, Yemeni isolates showed two sequence types, named TDRC1 and TDRC2, differing in a single nucleotide polymorphism. TDRC1 sequence type included 11 isolates that have microsatellite repeat numbers identical to those of *L*. *infantum* isolates from China and Mediterranean region. TDRC2 sequence type included 14 isolates that have microsatellite repeat numbers identical to those of *L*. *donovani* isolates from China and East Africa (Table 1).

**Table 1:** Microsatellite repeat numbers of ITS1 gene found for *L*. *donovani* complex isolates from Yemeni children infected with VL compared to reference strains.^a^

Strain/ isolate international code	Species	Origin	Poly C	Poly A	Poly TA	Poly A	GenBank accession No.
MHOM/FR/95/LPN114	*L*. *infantum*	France	3	6	4	8	AJ634340
MHOM/ES/93/PM1	*L*. *infantum*	Spain	3	6	4	8	AJ634341
MHOM/PT/00/IMT260	*L*. *infantum*	Portugal	3	6	4	8	AJ634344
MHOM/CN/54/Peking	*L*. *infantum*	China	3	6	4	8	AJ634345
MHOM/TN/80/IPT1	*L*. *infantum*	Tunisia	3	6	4	8	AJ000289
MHOM/IT/94/ISS1036	*L*. *infantum*	Italy	3	6	4	8	AJ634353
MHOM/YE/10/SH2, 4, 9, 15, 16, 17, 18, 22, 27, 28, 41^b^	*L*. *infantum*	Yemen	3	6	4	8	KT751245 to KT751255
MHOM/CN/00/Wangjie1	*L*. *donovani*	China	3	6	4	7	AJ000294
MCAN/SD/00/LEM3946	*L*. *donovani*	Sudan	3	6	4	7	AJ634356
MHOM/ET/00/HUSSEN	*L*. *donovani*	Ethiopia	3	6	4	7	AJ634360
MHOM/YE/10/SH3, 6, 7, 8, 10, 12, 14, 20, 21, 23, 29, 31, 37, 40^c^	*L*. *donovani*	Yemen	3	6	4	7	KT751256 to KT751269
MHOM/SD/62/LRC-L61	*L*. *donovani*	Sudan	2	8	6	8	AJ634365
MHOM/SD/68/1S	*L*. *donovani*	Sudan	2	8	6	8	AJ000293
MHOM/SD/75/LV139	*L*. *donovani*	Sudan	2	8	6	8	AJ000291
MHOM/IN/71/LRC-L51a	*L*. *donovani*	India	2	8	5	7	AJ000290
MHOM/IN/80/DD8	*L*. *donovani*	India	2	8	5	7	AJ000292
MHOM/KE/84/NLB218	*L*. *donovani*	Kenya	2	8	5	7	AJ000296
MHOM/SD/93/9S	*L*. *donovani*	Sudan	2	9	5	7	AJ634372
MHOM/ET/67/HU3	*L*. *donovani*	Ethiopia	2	9	5	7	AJ634373

^a^ Reference strains are published by Kuhls et al. [18];

^b^ isolates with TDRC1 sequence type in the present study;

^c^ isolates with TDRC2 sequence type in the present study

## Discussion

VL is the most severe form of leishmaniasis that is caused by the species of *L*. *donovani* complex (*L*. *donovani* and *L*. *infantum*). Although differentiation between these two species is important because of the differences in their epidemiology and pathology, this had been a difficult task. Typing this parasite using isoenzyme analysis is difficult and requires a large amount of the parasite [27,28] or may sometimes be unreliable [29]. The advent of molecular tools provided methods which are robust in discriminating these two species and to study their phylogenetic relationships [18,30–32]. Although pediatric VL among Yemeni children was clinically described [12,14], the molecular characterization and phylogenetic relationship among VL-causing *Leishmania* isolates had not been studied before. The present study deploys molecular approaches to characterize VL-causing *Leishmania* species from clinical isolates collected from bone marrows of Yemeni children based on the analysis of ribosomal ITS1 sequences.

*L*. *donovani* complex causing VL among Yemeni children exhibited a monophyletic cluster that is consistent with *L*. *infantum* isolates from Europe and China as well as *L*. *donovani* isolates from Africa. This finding is in agreement with that of Mauricio et al. [33], where phylogenetic trees generated by the combined data of five genes of *L*. *donovani* complex could not give a clear division between *L*. *infantum* and *L*. *donovani*. Hence, this finding strongly supports early hypotheses of the monophyletic origin of *Leishmania* species from the Old World and the New World based on zymodeme analysis [34] and DNA-based characterization [35]. The finding is also consistent with the monophyletic relationship among Turkish isolates of *L*. *infantum* recently reported in comparison to *L*. *tropica* that showed different phylogenetic groups based on ITS1 sequence [36]. In contrast, Yang et al. [37] reported the heterogeneity of Chinese *Leishmania* isolates and suggested that they do not form a monophyletic group based on the sequence analysis of ITS1, which was then confirmed by sequencing of kinetoplast cytochrome oxidase II gene [38]. The phylogenetic tree constructed from the sequences of the present study isolates and sequences from *L*. *donovani* complex from other parts of the world shows that Yemeni isolates are genetically similar to them.

Based on the analysis of the four polymorphic microsatellites in the ITS1 sequences of *L*. *donovani* complex in the present study, isolates showed two distinct alleles or sequence types (TDRC1 and TDRC2) differing in a single nucleotide polymorphism. The ITS1 sequence types of 11 isolates were similar to those of *L*. *infantum* strain zymodemes originating from different geographical regions, with the exception of *L*. *infantum* zymodemes from Sudan (MON-30, 81, 267) [18]. However, these Sudanese zymodemes were later regarded as *L*. *donovani* rather than *L*. *infantum* based on microsatellite analysis and that *L*. *donovani* is the only agent causing VL in East Africa [18,29,39,40]. In general, phylogenetic trees generated from DNA-based analyses might not correlate with isoenzyme-based taxonomy.

These 11 isolates with TDRC1 sequence type showed the same sequence types at the four polymorphic microsatellites as those reported by Kuhls et al. [18] from France (MON-1, 11, 29, 108), Spain (MON-1, 77, 183, 198, 199), Portugal (MON-1), Italy (MON-188, 228), Malta (MON-78), China (MON-1 and LON-49) and Tunisia (MON-1). Sequence similarity also exists with MON-1 zymodeme of *L*. *chagasi* from Brazil [18]. It is important to consider that *L*. *chagasi* has been regarded as a synonym of *L*. *infantum* based on DNA analysis by a variety of techniques [18,41,42]. It is noteworthy to highlight that *L*. *infantum* differs from *L*. *donovani* with regards to its ITS1 microsatellite repeat numbers as shown in the study by Kuhls et al. [18]. This strongly supports the existence of *L*. *infantum* as a causative agent of VL among Yemeni children based on molecular sequencing. Moreover, reporting canine leishmaniasis and that dogs possibly act as reservoir hosts for VL [12] further supports the possible role of *L*. *infantum* as a causative agent of VL in Yemen.

On the other hand, 14 of the studied isolates with TDRC2 sequence type are similar to those of *L*. *donovani* zymodemes originating from Old World countries; namely, Sudan (MON-274), Ethiopia (MON-31) and China (MON-35) as well as those of *L*. *archibaldi* zymodemes from Sudan (MON-257, 258) [18]. *L*. *archibaldi* is a disputed species that has been suggested to be a synonym of *L*. *donovani* based on DNA analysis by a variety of techniques [31,40,41]. However, ITS1 sequences of Yemeni isolates differ from those of *L*. *donovani* zymodemes from India (MON-2, 38), Kenya (MON-274), Sudan and Ethiopia (MON-18) [18]. Sequence differences of the latter 14 isolates from sequences of well-characterized *L*. *infantum* and their similarity to well-characterized *L*. *donovani* strongly suggest that these isolates are *L*. *donovani*. In addition to its small sample size, another limitation of this study is that it did not consider ITS2 sequencing. However, according to Schonian et al. [32], sequencing of the ITS1 allows the differentiation of the Old World *L*. *donovani* complex, with a clear distinction of *L*. *infantum* from *L*. *donovani*. Moreover, beyond species identification, ITS1 sequencing enables the assignment of *L*. *donovani* complex strains to different phylogenetic groups supported by their biological characteristics and clinical outcomes [18]. With the presence of *L*. *donovani* and *L*. *infantum* in the country, there is a need to determine the relative contribution of both parasite species to the anthroponotic and/or zoonotic epidemiology of VL.

Questions remain unanswered include whether dogs and other animals are possible reservoir hosts and whether *L*. *donovani* has an anthroponotic transmission similar to that in the Indian subcontinent. The detection of circulating antibodies against *Leishmania* species in sera of dogs in Yemen [12] raises concerns about the possible role of zoonotic transmission from dogs to humans. Postigo [6] reported the occurrence of zoonotic VL caused by *L*. *infantum* in most countries of the EMR of the WHO, including Yemen. In contrast to the anthroponotic CL in Yemen, the anthroponotic VL caused by *L*. *donovani* remains unclear yet probable in the country [6]. The presence of *L*. *donovani* and *L*. *infantum* necessitates further investigation for the nature of transmission and the possible involvement of animal reservoir hosts in their transmission to tailor appropriate prevention and control strategies.

In conclusion, the present study reveals that both *L*. *infantum* and *L*. *donovani* are causative agents of pediatric VL in Yemen in line with previous characterization using isoenzyme analysis. Therefore, both anthroponotic and zoonotic epidemiological patterns of transmission could not be ruled out. For effective control, determination of possible animal reservoir hosts should be explored using molecular methods.
